# Melatonin receptor expression in *Xenopus laevis* surface corneal epithelium: Diurnal rhythm of lateral membrane localization

**Published:** 2009-11-17

**Authors:** Allan F. Wiechmann, Lindsey R. Hollaway, Jody A. Summers Rada

**Affiliations:** 1 Department of Cell Biology, University of Oklahoma Health Sciences Center, Oklahoma City, OK; 2 Department of Ophthalmology, University of Oklahoma Health Sciences Center, Oklahoma City, OK; 3 Oklahoma Center for Neuroscience, University of Oklahoma Health Sciences Center, Oklahoma City, OK

## Abstract

**Purpose:**

Melatonin receptors are seven-pass G protein-coupled receptors located in many tissues throughout the body, including the corneal epithelium (CE), and relay circadian signals to the target cells. The purpose of this study was to determine more precisely the cellular distribution of the melatonin receptors in the surface cells of the CE of *Xenopus laevis*, and to examine the relative distribution of melatonin receptor subtype expression at different times during the circadian cycle.

**Methods:**

Cryostat sections and whole corneas of adult *Xenopus laevis* were processed for immunocytochemistry using antibodies specific for each of the three melatonin receptor subtypes (Mel1a, Mel1b, and Mel1c). For the circadian studies, corneas were obtained from euthanized frogs at 4-h intervals during a 24-h period under a 12 h:12 h light-dark cycle. Double-label immunocytochemistry was performed using a Mel1a antibody in combination with antibodies against Mel1b, Mel1c, or the zonula occludens protein ZO-1. Corneal whole-mount specimens and corneal sections were analyzed by laser-scanning confocal microscopy.

**Results:**

All three melatonin receptor subtypes were expressed on the surface and sub-superficial layer of CE cells, but with different sub-cellular distributions. The Mel1a receptor was highly localized to the lateral plasma membrane of the surface CE, but also displayed cytoplasmic localization at some times of day, especially at night. Mel1c showed a similar pattern of labeling to Mel1a, but there were some distinctive differences, insofar as the Mel1c receptors were usually located immediately basal to the Mel1a receptors. The relative degree of membrane and cytoplasmic labeling of the Mel1c receptor also oscillated during the 24-h period, but was out of phase with the changes that occurred in the Mel1a receptor localization. Furthermore, in the late afternoon time point, the Mel1a and Mel1c receptors were highly co-localized, suggestive of heterodimerization, whereas at other time points, the two receptors were distinctly not co-localized. Double-label immunocytochemistry of Mel1a and ZO-1 demonstrated that the Mel1a receptor was located basal to the tight junctions, on the lateral membrane in very close proximity to the ZO-1 protein.

**Conclusions:**

Mel1a, Mel1b, and Mel1c receptor subtypes are expressed in the lateral plasma membrane of the *Xenopus* surface CE, at a position in close proximity to the tight junctions that form the corneal diffusion barrier. The very close association of the Mel1a receptors to the ZO-1 peripheral membrane tight junction proteins is suggestive of a potential role for melatonin in influencing the rate of tight junction formation or breakdown. The transient co-localization of Mel1a and Mel1c late in the light period is suggestive of formation of heterodimers that may influence receptor responsiveness and/or activity during specific periods of the day. The dynamic daily changes in melatonin receptor subtype expression and localization in the surface CE supports the concept that melatonin signaling may affect circadian activities of the surface epithelium of the cornea.

## Introduction

Melatonin receptors are located throughout the body, including many ocular tissues, presumably to mediate the effects of nighttime melatonin on circadian activities [1]. Melatonin is a circadian signaling molecule produced at night time by the pineal gland, retinal photoreceptors, and ciliary epithelium [2-6]. Melatonin receptors are G protein-coupled seven-pass transmembrane receptors, and are expressed in the corneal epithelium (CE) [7-9], but their functions are unknown, and the precise location of the three receptor subtypes on the CE is not known. The turnover of surface CE cells is thought to occur on a daily basis, but the mechanism of how this occurs is poorly understood [10,11]. Furthermore, the CE cells that are directly underneath the surface may require a circadian signal to pre-accumulate the proteins needed to quickly re-establish the CE permeability barrier after the surface cells are shed [12,13].

The balance in the rate of corneal epithelium proliferation and desquamation is crucial for maintenance of corneal health and function, and these processes appear to undergo changes on a daily basis [10,11,14-19]. Temporal coordination of desquamation of the surface epithelium and subsequent formation of the new tight junction barrier by the underlying cells may perhaps be facilitated by circadian signals such as melatonin. To investigate the possibility that melatonin signaling may have a role in the circadian activities of corneal epithelial cells, the cellular distribution of Mel1a, Mel1b, and Mel1c melatonin receptor subtype proteins in the *Xenopus laevis* CE was examined by confocal immunocytochemistry.

## Methods

### Animals and tissue processing procedures

Post-metamorphic *Xenopus laevis* (African clawed frogs) were obtained from Xenopus 1 (Dexter, MI) and maintained in aquaria at 20 °C on a daily 12 h:12 h light–dark schedule (lights on: 6:00 AM; lights off: 6:00 PM). Frogs were anesthetized by immersion in tricaine methanesulfonate (MS-222) and killed by decapitation. Tissues were fixed for 18 hr at 4 °C in 4% paraformaldehyde in 0.1 M phosphate buffer, pH 7.4. Corneas were dissected from the eyes and rinsed with 0.1 M phosphate-buffered saline (PBS), pH 7.4. For immunocytochemistry of cryostat sections, corneas were transferred to 30% sucrose in phosphate buffer for 16–20 h at 4 °C, and then mounted in Tissue-Tek O.C.T. mounting matrix (Sakura Finetek, Torrance, CA). Sagittal 10 μm sections were cut on a cryostat microtome and collected on glass slides. For whole mount immunocytochemistry, corneas were placed separately into 2.0 ml microfuge tubes and processed for immunocytochemistry. The animals were cared for in accordance with the guidelines of the Public Health Service Policy on Humane Care and Use of Laboratory Animals.

### Confocal immunocytochemistry procedures

For immunocytochemical localization of melatonin receptors in *Xenopus laevis* CE, cryostat sections or whole corneas were rinsed in PBS, and then incubated in incubation buffer (2% bovine serum albumin [Sigma, St Louis, MO], 0.2% Triton X-100, and 0.004% sodium azide in PBS) for 30 min at room temperature (RT). Sections were incubated either with chicken anti-*Xenopus* Mel1a melatonin receptor antibody, which has been previously characterized as specific for the *Xenopus* Mel1a receptor subtype [20], rabbit anti-*Xenopus* Mel1b melatonin receptor antibody, which has been previously characterized as specific for the *Xenopus* Mel1b receptor subtype [21], or rabbit anti-*Xenopus* Mel1c melatonin receptor antibody, which has been previously characterized as specific for the *Xenopus* Mel1c receptor subtype [22].

The affinity-purified melatonin receptor antibodies were used at a concentration of 2.3 µg/ml in incubation buffer, and were incubated with the sections or whole corneas for 3 days at 4 °C. For negative controls, tissue sections were incubated in incubation buffer lacking the primary antibody. Following incubation with the primary antibody, sections or whole corneas were rinsed in PBS, and incubated in 5 μg/ml of goat anti-chicken antibody or goat anti-rabbit antibody conjugated to AlexaFluor 488 (green; Molecular Probes, Eugene, OR) for 1 h at RT. Sections were rinsed in PBS, then incubated with 0.0005% 4´, 6-diamidino-2-phenylindole (DAPI: Invitrogen, Carlsbad, CA) nuclear stain for 10 s at RT, followed by a final rinse in PBS. Corneal whole mounts were incubated in 0.0005% DAPI for 10 min at RT, followed by a rinse in PBS. Corneas were mounted onto glass slides by making 4–5 slits from peripheral to central cornea with scissors and then compressing the tissue under the coverslips after the mounting matrix was applied to achieve a flat-mounted cornea. Coverslips were mounted onto the slides with Prolong Gold antifade reagent containing DAPI (Invitrogen).

For double-label immunocytochemistry, the same procedure was followed as described for the primary antibody incubations, except that the first primary antibody was the Mel1a melatonin receptor antibody, which was subsequently labeled with 5 μg/ml of goat anti-chicken antibody as a secondary antibody, conjugated with AlexaFluor 568 (red; Molecular Probes). Following the rinse in PBS after the 1-h secondary antibody incubation, the sections or whole corneas were incubated in either rabbit anti-*Xenopus* Mel1b or rabbit anti-*Xenopus* Mel1c melatonin receptor antibodies (2.3 µg/ml) or mouse anti-human ZO-1 antiserum (Zymed, San Francisco, CA) at a dilution of 1:25 for 3 days at 4 °C. Following incubation with the second primary antibody, sections or whole corneas were rinsed in PBS, and incubated in 5 μg/ml of goat anti-rabbit antibody or goat anti-mouse antibody conjugated to AlexaFluor 488 (green) (Molecular Probes) for 1 h at RT. Sections were rinsed in PBS, then incubated with 0.0005% DAPI nuclear stain for 10 s at RT, followed by a final rinse in PBS. Whole corneas were incubated in 0.0005% DAPI for 10 min at RT, followed by a rinse in PBS. Coverslips were mounted onto the slides with Prolong Gold antifade reagent containing DAPI. The immunolabeled sections and corneal whole mounts were viewed by confocal microscopy, using an Olympus FluoView 1000 laser-scanning confocal microscope (Olympus, Center Valley, PA). The pinhole (confocal aperture diameter) conditions were fixed at 105 µm in all images generated in this study. The objective lens used in this study was an Olympus PlanApo N 60x/1.42 Oil lens (∞/0.17/FN26.5). The control specimens were always examined under identical conditions to the appropriate non-control specimens.

## Results

### Melatonin receptor localization in corneal cryostat sections

**Figure 1:**
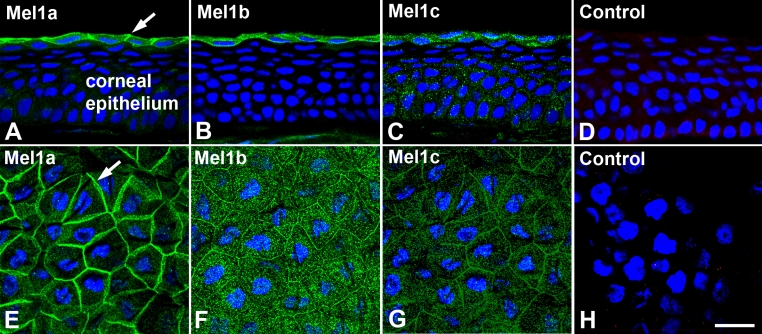
Mel1a, Mel1b, and Mel1c immunocytochemistry of cryostat sections and whole mounts of *Xenopus laevis* corneal epithelium. **A**-**C**: Cryostat sections of corneas obtained during the light period were immunolabeled with Mel1a, Mel1b, or Mel1c receptor antibodies. Arrows in panel A indicate the immunolabeled plasma membranes of the surface epithelium. **D**: The control specimen was processed in the absence of primary antibody. **E-G**: Whole mount preparations of corneas obtained during the light period were immunolabeled with Mel1a, Mel1b, or Mel1c receptor antibodies. **H**: The control whole mount specimen was processed in the absence of primary antibody. Primary antibodies were labeled with secondary antibody conjugated to AlexaFluor 488 (green fluorescence). Most of the Mel1a receptor labeling (**A** and **E**) occurs in the lateral plasma membrane of the surface epithelium, whereas there is a higher proportion of Mel1b (**B** and **F**) and Mel1c (**C** and **G**) labeling also present in cytoplasmic compartments in addition to the lateral membranes. Note that no specific immunolabeling is detected in the control specimens (**D** and **H**). Nuclei are stained with DAPI. The magnification bar (**H**) represents 20 µm.

Mel1a receptor immunoreactivity in *Xenopus* CE was observed to be localized to the surface layer of cells in sagittal sections (Figure 1A). In whole mount corneas, the Mel1a immunoreactivity of the surface CE was most intense in the plasma membranes (Figure 1E). Mel1b receptor immunoreactivity was also localized to the surface layer of cells in sagittal sections (Figure 1B). In whole mount corneas, although there was immunoreactivity present in the lateral plasma membranes, there was also a considerable amount of immunoreactivity in small cytoplasmic compartments (Figure 1F). Similarly, Mel1c receptor immunoreactivity was localized mostly to the surface layer of cells in sagittal sections, although some punctate immunolabeling was also observed in the more basal layers of the CE (Figure 1C). In whole mount corneas, the Mel1c immunoreactivity of the surface CE was most intense in the lateral plasma membranes, but there was also some immunolabeling of cytoplasmic compartments (Figure 1G). Sections and whole corneas treated without primary antibody were devoid of specific immunoreactivity (Figures D, H). These observations suggest that there is a differential expression of the three melatonin receptor subtypes in the *Xenopus* CE.

**Figure 2:**
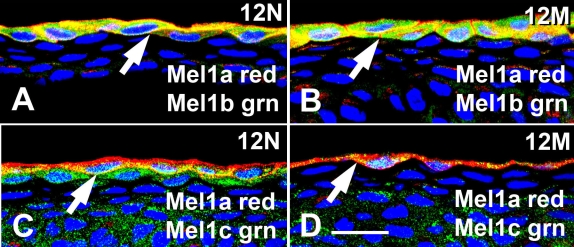
Mel1a, Mel1b, and Mel1c double-label immunocytochemistry of cryostat sections of *Xenopus laevis* corneal epithelium. **A** and **C**: Corneas obtained at 12:00 noon (12N) in the light. **B** and **D**: Corneas obtained at 12:00 midnight (12M) in the dark. Sections were immunolabeled with Mel1a and either Mel1b or Mel1c receptor antibodies. Mel1a labeling is represented in red, and Mel1b and Mel1c labeling is represented in green. Yellow indicates regions of co-localization of the red and green signal. Melatonin receptors are expressed in the surface epithelium, but their relative levels of expression and distribution change between 12N and 12M. Arrows indicate the immunolabeled plasma membranes of the surface epithelium. Nuclei are stained with DAPI. The magnification bar (**D**) represents 20 µm.

Double-labeled cryostat sections of *Xenopus* CE demonstrated both a co-localization and differential distribution of Mel1a, Mel1b, and Mel1c melatonin receptor immunoreactivity at the mid-light (12N) and mid-dark (12M) time points (Figure 2). Since the Mel1a antibody was raised in chickens, and the Mel1b and Mel1c antibodies were raised in rabbits, the only combinations of receptor immunocytochemical double labeling that were feasible were the combinations of Mel1a with Mel1b, and Mel1a with Mel1c. At the mid-light time point (12N), the red Mel1a immunolabeling was observed in the plasma membrane, and the green immunoreactivity of the Mel1b label was observed in the cytoplasm (Figure 2A). The region of the cells that are labeled yellow indicates co-localization of the Mel1a and Mel1b, insofar as both receptors appeared to be located on the plasma membrane (Figure 2A). At the mid-dark (12M) time point, the pattern of both Mel1a and Mel1b immunoreactivity appeared very similar to the 12N time point in these cryostat sections (Figure 2B).

At the mid-light time point (12N), the red Mel1a immunolabeling was again observed in the plasma membrane and cytoplasm, and the green immunoreactivity of the Mel1c label was observed not only in the cytoplasm of the surface layer of CE, but also in the sub-superficial layer of cells (Figure 2C). Again, the region of the cells that were labeled yellow indicates a close proximity or co-localization of the Mel1a and Mel1c labels. At the mid-dark (12M) time point, Mel1a (red) was present in the surface layer of cells, but Mel1c immunoreactivity (green) appeared to be greatly reduced in these cells and in the sub-superficial cells, insofar as there appeared to be only a small amount of yellow punctate immunoreactivity in the surface cell cytoplasm in these cryostat sections (Figure 2D). These observations suggest that there is a translocation and/or change in expression of some of the melatonin receptor subtypes between the light and dark period, and/or turnover of surface CE cells.

### Mel1a and Mel1c receptor localization in whole corneas

**Figure 3:**
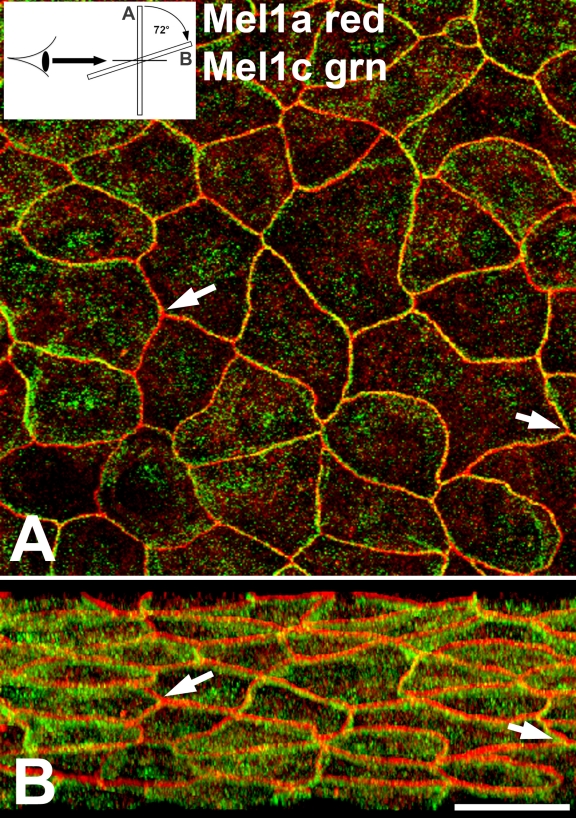
Confocal double-label immunocytochemical localization of Mel1a and Mel1c in *Xenopus* corneal whole mounts. **A**: The specimen shown was obtained in the mid-light period (12N). Both Mel1a and Mel1c immunolabeling is observed on the lateral plasma membrane, with some immunoreactivity also occurring in the cytoplasm. The labeling of the plasma membrane displays distinct areas of Mel1a (red), Mel1c (green), and both receptors (yellow). Arrows are provided as reference points to indicate the same points on **B**. The inset illustrates the 72° rotation on the x-axis of the image in **A**, indicating the orientation relative to the viewer’s eye in **B**. **B**: Three-dimensional reconstructions of confocal z-stacks of optical slices were rotated at 72° degrees on the x-axis to enable optimal viewing of the pattern of immunolabeling. The rotated image shows that the red Mel1a labeling is generally located apically to the green Mel1c labeling. The Mel1a labeling is seen as a relatively broad continuous band of red label on the lateral plasma membrane of the majority of surface CE cells. A somewhat broader band of green Mel1c labeling appears directly basal to the Mel1a label. Some yellow labeling is occasionally observed, indicating some co-localization of Mel1a and Mel1c. There are many areas in the red Mel1a band in which yellow labeling is interspersed between areas of red Mel1a labeling, suggesting that some green Mel1c-labeled receptor is interdigitated among the Mel1a-labeled receptor. The confocal images in both panels are comprised of 13 optical slices of 400 nm each in the z-series. The magnification bar (**B**) represents 20 µm.

A more detailed analysis of corneal whole mounts that were double-labeled with Mel1a and Mel1c antibodies was performed to determine the relative locations of the two receptor subtypes in the surface CE cells. In a typical mid-light (12N) specimen, both Mel1a and Mel1c immunolabeling was observed on the lateral plasma membrane, with some immunoreactivity also occurring in the cytoplasm (Figure 3). The labeling of the plasma membrane was characterized by a pattern of distinct areas of Mel1a (red), Mel1c (green), and both receptors (yellow; Figure 3A).

Three-dimensional reconstructions of confocal z-stacks of optical slices were rotated at 72° on the x-axis to enable optimal viewing of the pattern of immunolabeling. The rotated image demonstrated that the red Mel1a labeling was generally located apically to the green Mel1c labeling (Figure 3B). The pattern of Mel1a labeling was characterized by a relatively broad continuous band of red label on the lateral plasma membrane of the majority of surface CE cells. In many instances, a somewhat broader band of green Mel1c labeling appeared directly basal to the Mel1a label, although there were some areas in which the green Mel1c labeling was lacking or diminished. Some yellow labeling was observed, indicating the very close proximity of the red Mel1a and green Mel1c immunolabeling. Furthermore, there were many areas in the red Mel1a band in which some yellow labeling was interspersed between areas of red Mel1a labeling, suggesting that some green Mel1c-labeled receptors were present in the area of the lateral membrane that contained mostly Mel1a-labeled receptors.

**Figure 4:**
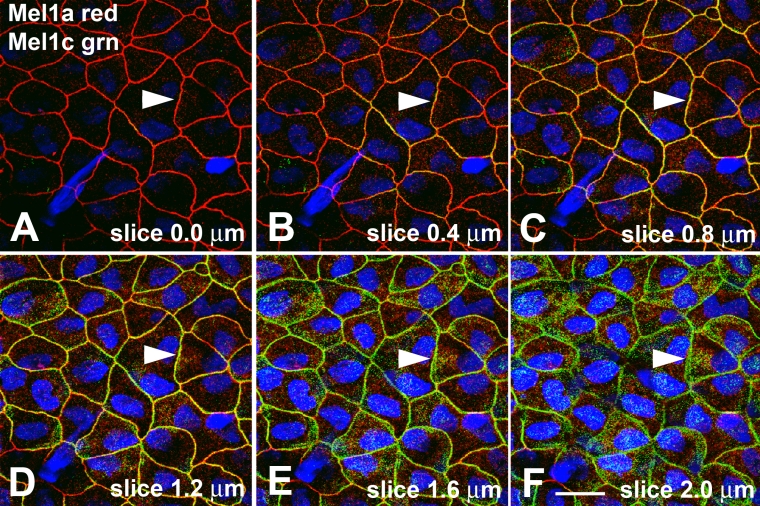
Localization of Mel1a and Mel1c in progressive confocal optical slices of *Xenopus* corneal epithelium. **A**: Image of the most superficial surface of the surface corneal epithelium. Note that only the red Mel1a immunoreactivity is present on the lateral membranes. **B-F**: As the 0.4 µm slices progress deeper into the corneal epithelium layer, the Mel1a immunoreactivity lessens, whereas the green Mel1c immunoreactivity increases (note arrowheads indicating an example of this), indicating that the Mel1c receptor is located basal to the Mel1a receptor. Nuclei are stained with DAPI. The magnification bar (**F**) represents 20 µm.

For further confirmation that the Mel1a labeling was located apically to the Mel1c immunolabeling, individual 400-nm optical slices were analyzed for their relative amounts of Mel1a and Mel1c immunolabeling (Figure 4). In the most apical slice (slice 0.0 µm), red Mel1a labeling was observed prominently (Figure 4A), but almost no green or yellow labeling was displayed. Progressing basally through the 400-nm optical slices, yellow immunolabeling became more prominent (Figure 4B-D) with concomitant decreases in red Mel1a labeling. The green Mel1c labeling then became increasingly prominent (Figure 4D-F), so that by optical slice 2.0 µm (Figure 4F), almost all plasma membrane labeling was due exclusively to the green Mel1c label. Most of the punctate green Mel1c label that was observed interior to the plasma membranes (Figure 4F) appeared to be due to Mel1c labeling of the lateral membrane as it flared obliquely between adjacent cells. So, some labeling that may initially appear as perhaps being cytoplasmic labeling is actually labeling of the lateral membranes that are oriented at an oblique angle.

### Mel1a and Mel1b receptor localization in whole corneas

**Figure 5:**
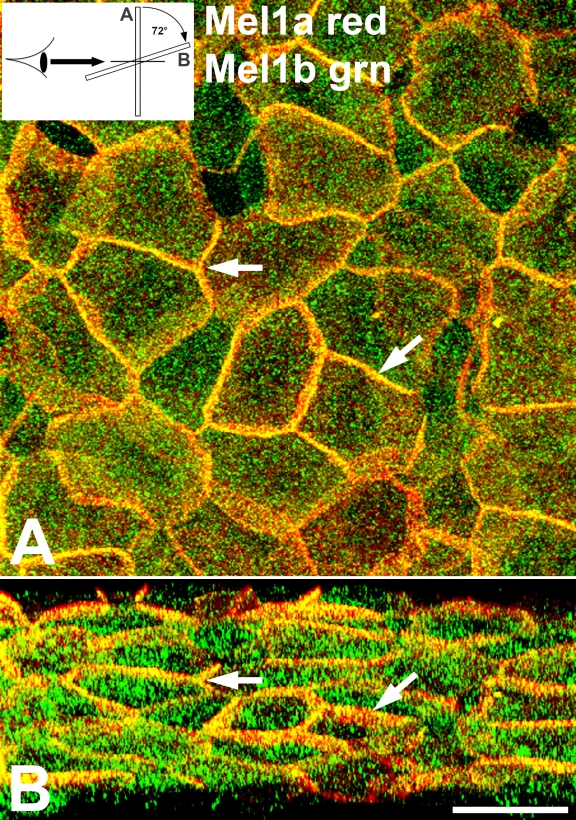
Confocal double-label immunocytochemical localization of Mel1a and Mel1b in *Xenopus* corneal whole mounts. **A**: The specimen shown was obtained in the mid-light period (12N). Both Mel1a (red) and Mel1b (green) immunolabeling is present on the lateral plasma membrane appearing mostly as the merged yellow fluorescence indicative of co-localization. A significant amount of green Mel1b immunoreactivity is also present in the cytoplasm. Arrows are provided as reference points to indicate the same points on panel B. The inset illustrates the 72° rotation on the x-axis of the image in **A**, indicating the orientation relative to the viewer’s eye in **B**. **B**: Three-dimensional reconstructions of confocal z-stacks of optical slices were rotated at 72° degrees on the x-axis to enable optimal viewing of the pattern of immunolabeling. The rotated image shows that the Mel1a-Mel1b-labeled cells are characterized by a broad band of merged yellow labeling, interdigitating with a lesser amount of red Mel1a labeling. This pattern of labeling suggests that a majority of Mel1a and Mel1b receptors are located in very close proximity to each other on the lateral membrane. The confocal images in both panels are comprised of 19 optical slices of 400 nm each in the z-series. The magnification bar (**B**) represents 20 µm.

An analysis of corneal whole mounts that were double-labeled with Mel1a and Mel1b antibodies was performed in a manner identical to those performed on the Mel1a-Mel1c double-labeled specimens. In a typical mid-light (12N) specimen, both Mel1a and Mel1b immunolabeling was observed on the lateral plasma membranes, but with a significant amount of Mel1b immunoreactivity also occurring in the cytoplasm (Figure 5A). However, the pattern of Mel1a-Mel1b labeling was significantly different from what was observed for the Mel1a-Mel1c labeling pattern.

The Mel1a-Mel1c immunolabeling (Figure 3) was characterized by a relatively broad continuous band of Mel1a on the lateral membrane of the majority of surface cells, with a somewhat broader, less defined band of green Mel1c labeling directly basal to the Mel1a label. In contrast, the Mel1a-Mel1b labeled cells were characterized by a broad band of merged yellow labeling, interdigitating with a lesser amount of red Mel1a labeling (Figure 5B). This pattern of labeling suggests that a majority of Mel1a and Mel1b receptors are located in very close proximity to each other on the lateral membrane, with a lesser amount of Mel1a that is not juxtaposed to the Mel1b receptor. Essentially all the green Mel1b labeling (that did not contribute to the merged yellow lateral membrane labeling) was present in cytoplasmic compartments, with most or all of the Mel1b labeling of the lateral membranes merged with the red Mel1a label to create the merged yellow labeling of the membrane. Therefore, whereas the Mel1c labeling was located basally to the Mel1a label (Figure 3B), the Mel1b label had the same cellular apical/basal position as the Mel1a label (Figure 5B).

**Figure 6:**
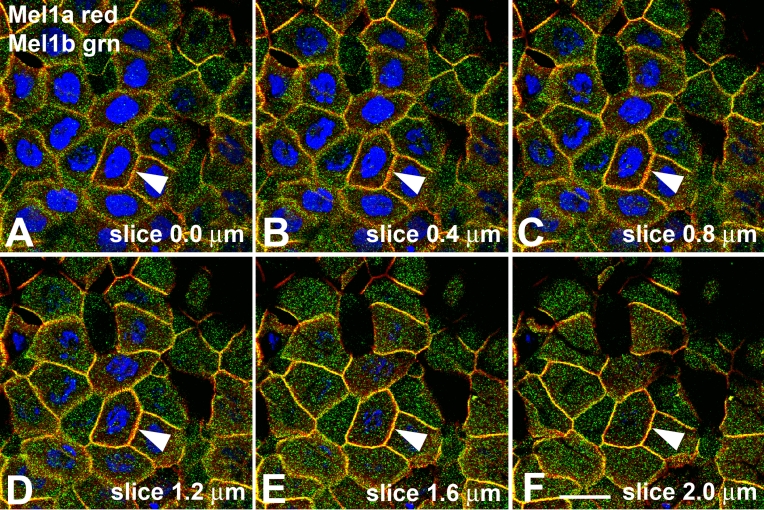
Localization of Mel1a and Mel1b in progressive confocal optical slices of *Xenopus* corneal epithelium. **A**: Image of the most superficial surface of the surface corneal epithelium. Note the predominance of yellow (merged red and green) labeling of most lateral membranes, with a lesser amount of interdigitated red Mel1a labeling (note arrowheads indicating an example of this). **B-F**: As the 0.4-µm slices progress deeper into the corneal epithelium layer , there is not a transition from red to green labeling as was seen with Mel1a-Mel1c, but instead the predominance of yellow labeling with some interspersed red labeling is maintained throughout all slices. Nuclei are stained with DAPI. The magnification bar (**F**) represents 20 µm.

To further confirm that the Mel1a and Mel1b receptors were located at the same apical/basal position, individual 400-nm optical slices were analyzed for their relative amounts of Mel1a and Mel1b immunolabeling (Figure 6), as was done for the Mel1a-Mel1c labeling in Figure 4. In all slices, there was not a transition from red to green labeling, but instead a predominance of yellow labeling with some interspersed red labeling (Figure 6). This is in contrast to the Mel1a-Mel1c pattern of labeling, in which there was a distinctive transition from red Mel1a to green Mel1c labeling (Figure 4). This analysis confirmed that the Mel1a and Mel1b receptors have a spatial relationship distinct from that of the Mel1a and Mel1c receptors.

### Mel1a receptor and ZO-1 localization in whole corneas

An analysis of corneal whole mounts that were double-labeled with Mel1a and ZO-1 antibodies was performed in a manner identical to those performed on the Mel1a-Mel1c double-labeled specimens. ZO-1 is a cytoplasmic protein that binds directly to integral membrane proteins of zonula occludens, and is therefore a marker for tight junctions. Double-label immunocytochemistry with antibodies to Mel1a and to ZO-1 were performed to determine the relative location of the melatonin receptors to tight junctions on the lateral membranes of CE cells.

**Figure 7:**
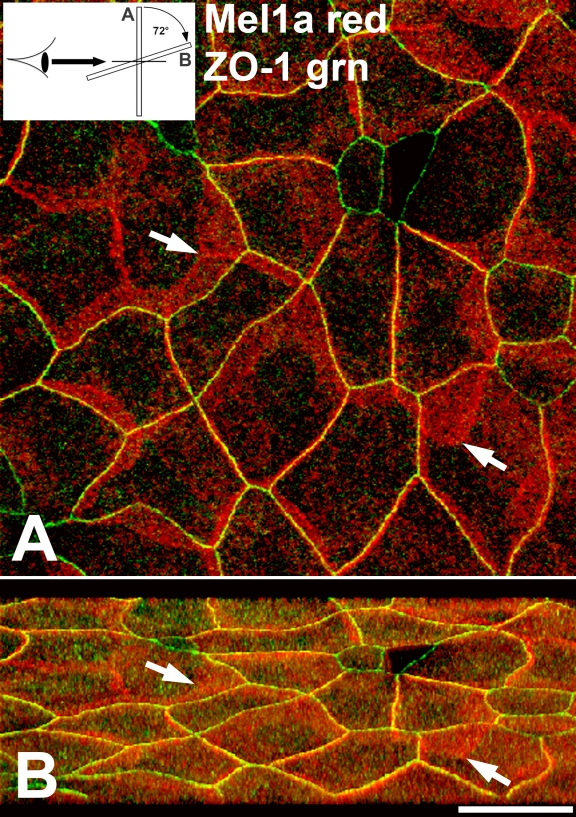
Confocal double-label immunocytochemical localization of Mel1a and ZO-1 in *Xenopus* corneal whole mounts. **A**: Mel1a and ZO-1 immunolabeling is observed on the lateral plasma membrane, with some immunoreactivity also occurring in the cytoplasm. Mel1a (red) labeling is present predominantly as very broad bands on the lateral membranes, including the obliquely-oriented lateral membranes (arrows). The plasma membrane has an abundance of yellow labeling, indicative of a very close proximity of red Mel1a and green ZO-1. The ZO-1 labeling was not as broadly distributed on the lateral membrane. The inset illustrates the 72° rotation on the x-axis of the image in **A**, indicating the orientation relative to the viewer’s eye in **B**. **B**: Three-dimensional reconstructions of confocal z-stacks of optical slices were rotated at 72° degrees on the x-axis to enable optimal viewing of the pattern of immunolabeling. Arrows are provided as reference points to indicate the same points on panel **A**. The rotated image shows that the green ZO-1 labeling is generally located apically to the red Mel1a labeling. The ZO-1 labeling appears as a relatively narrow continuous band of green labeling on the lateral plasma membrane, whereas a much broader band of red Mel1a labeling appears directly basal to the ZO-1 label. Significant yellow labeling is observed, indicating that the Mel1a receptor is in very close proximity to ZO-1. The confocal images in both panels are comprised of seven optical slices of 400 nm each in the z-series. The magnification bar (**B**) represents 20 µm.

In a typical light-adapted (4PM) specimen, both Mel1a and ZO-1 immunolabeling was observed on the lateral plasma membrane, with some immunoreactivity also occurring in the cytoplasm (Figure 7). This is representative of what was observed at several other time points (data not shown). The Mel1a (red) labeling was present predominantly as very broad bands on the lateral membranes as described earlier, with a further observation that red Mel1a punctate labeling was abundant on obliquely-oriented lateral membranes (Figure 7A) as described earlier for Mel1a and Mel1c receptors. The plasma membrane was characterized by an abundance of yellow labeling, indicative of a very close proximity of Mel1a (red) and ZO-1 (green). The ZO-1 pattern of labeling was much more discreet than the Mel1a pattern, insofar as it was not as broadly distributed on the lateral membrane. Most of the plasma membrane labeling demonstrated the presence of both ZO-1 and Mel1a, but there were some areas in which only ZO-1 or only Mel1a were present. For example, cells that have very little apical surface membrane are assumed to be cells that have very recently reached the apical surface, and may not have yet formed fully-functional tight junction barriers; in many instances, these cells with small apical profiles expressed ZO-1 (green) labeling but a paucity of Mel1a (red) labeling (Figure 7A).

As described above for the analysis of Mel1a-Mel1c double-label experiments, three-dimensional reconstructions of confocal z-stacks of optical slices were rotated at 72° on the x-axis to enable optimal viewing of the pattern of immunolabeling. The rotated image demonstrated that the green ZO-1 labeling was generally located apically to the red Mel1a labeling (Figure 7B). The pattern of ZO-1 labeling was characterized by a relatively narrow continuous band of green label on the lateral plasma membrane of the majority of surface CE cells. A much broader band of red Mel1a labeling appeared directly basal to the ZO-1 label, although there were some areas in which the red Mel1a labeling was lacking or diminished. Significant yellow labeling was observed, indicating that the Mel1a receptor is in very close proximity to ZO-1. These observations indicate that Mel1a receptors are located on the lateral membranes basal to tight junctions in *Xenopus* surface CE cells.

**Figure 8:**
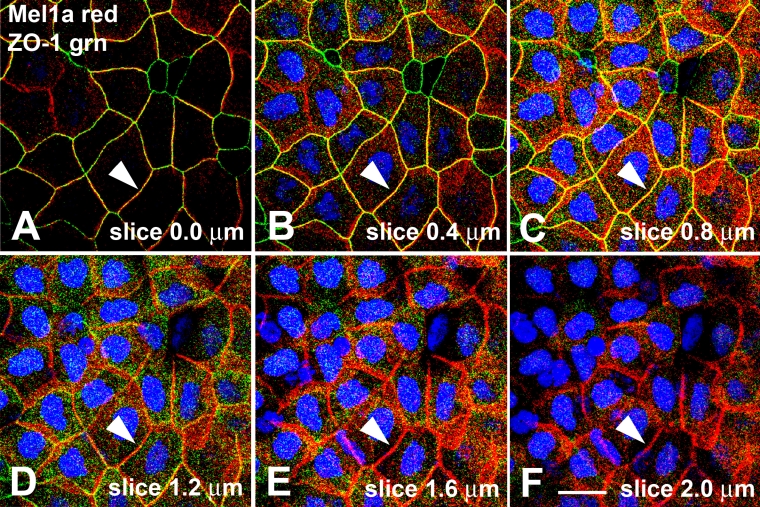
Localization of ZO-1 and Mel1a in progressive confocal optical slices of *Xenopus* corneal epithelium. **A**: Image of the most superficial surface of the surface corneal epithelium. In the most apical slice (slice 0.0 µm), red Mel1a, green ZO-1, and merged yellow labeling is observed in variable amounts on different cell membranes. **B-F**: As the 0.4-µm slices progress deeper into the corneal epithelium layer, the green ZO-1 immunoreactivity gradually lessens, whereas the red Mel1a immunoreactivity increases (note arrowheads indicating an example of this), indicating that the Mel1a receptor is located basal to the zonula adherens, but is in very close proximity in most of the slices (**A**-**C**). Nuclei are stained with DAPI. The magnification bar (F) represents 20 µm.

Analysis of individual 400-nm optical slices confirmed that the ZO-1 labeling was located apically to the Mel1a immunolabeling (Figure 8). In the most apical slice (slice 0.0 µm), red Mel1a, green ZO-1, and merged yellow labeling was observed (Figure 8A). This is in contrast to the almost exclusive localization of Mel1a labeling in the most apical optical slice described for the Mel1a-Mel1c double-labeling (Figure 3A). Progressing basally through the 400-nm optical slices, the red Mel1a, green ZO-1, and merged yellow persisted on the lateral membranes, with a general increase in the expression of the red Mel1a label (Figure 8B-D). Red Mel1c labeling continued to become increasingly prominent in the more basal slices (Figure 8D-F), so that by optical slice 2.0 µm (Figure 8F), essentially all the plasma membrane labeling was due to the red Mel1a label. Most of the punctate red Mel1a label that was observed interior to the plasma membranes (Figure 8B-F) appeared to be due to Mel1a labeling of the lateral membrane as it flared obliquely between adjacent cells. Some ZO-1 immunolabeling was observed in cytoplasm not associated with the plasma membrane, which is anticipated, since ZO-1 is known to have a cytoplasmic localization [23,24].

### Diurnal rhythm of melatonin receptor localization

Mel1a-Mel1c double-label immunocytochemistry was performed on whole corneas obtained from seven frogs at 4-h intervals during a 24-h period. The purpose was to determine if there were any observable changes in the expression or distribution of melatonin receptors in the surface CE that might potentially reflect diurnal or circadian changes in cellular responsiveness to melatonin. Frogs were housed under a 12 h:12 h light–dark cycle (6:00 AM: lights on; 6:00 PM: lights off).

**Figure 9:**
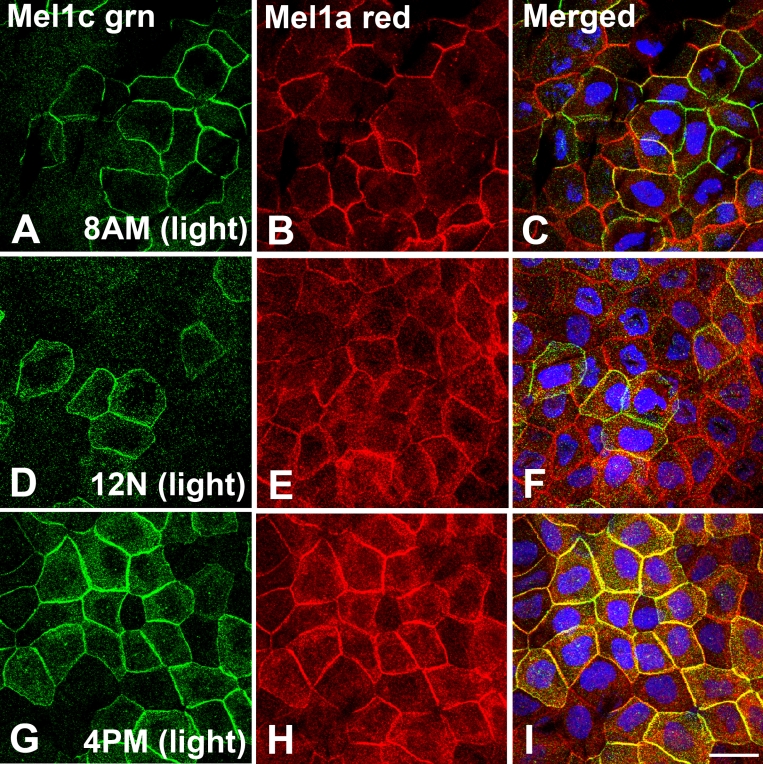
Mel1a and Mel1c immunocytochemistry of whole-mounted *Xenopus laevis* surface corneal epithelium obtained at 4-h intervals during a 24-h light–dark cycle. Frogs were housed under a 12 h:12 h light–dark cycle (6:00 AM: lights on; 6:00 PM: lights off). All tissues in this figure were obtained in the light. Mel1c labeling is represented in green (**A**, **D**, and **G**) and Mel1a labeling is represented in red (**B**, **E**, and **H**). The yellow labeling in the merged images (**C**, **F**, and **I**) indicates regions of co-localization of the red and green signal. **A-C**: Corneas obtained at 8:00 AM (2 h after lights on). Mel1c and Mel1a labeling is localized to the lateral plasma membrane of different yet overlapping subpopulations of cells. **D-F**: Corneas obtained at 12:00 N (mid-light). Mel1a and Mel1c immunolabeling is present on the lateral membranes of different populations of CE cells, with some minor regions of overlap. **G-I**: Corneas obtained at 4:00 PM (2 h before lights off). Most of the Mel1a and Mel1c immunolabeling is co-localized on the lateral membranes, with some cytoplasmic labeling that is not co-localized. Nuclei are stained with DAPI. The confocal images in all panels are comprised of three optical slices of 400 nm each in the z-series. The magnification bar (**I**) represents 20 µm.

At 8:00 AM (2 h after lights on), Mel1c (green) labeling was localized to the lateral plasma membrane of a subpopulation of CE cells (Figure 9A). Mel1a (red) labeling was also observed on the lateral membranes of a subpopulation of CE cells (Figure 9B). Of these two subpopulations of cells, some cells expressed only Mel1a or only Mel1c, whereas other cells expressed both Mel1a and Mel1c (Figure 9C). Furthermore, in most instances, the receptor immunolabeling was not uniform throughout the cell. A common pattern of receptor distribution was a dominance of one receptor subtype on a portion of the lateral membrane, and a dominance of the other receptor subtype on a different portion of the membrane.

At 12:00 N (noon; middle of the light period), Mel1c (green) immunolabeling was present in the lateral membranes of a subpopulation of CE cells, and Mel1a labeling (red) in another subpopulation of cells (Figure 9D-F). In contrast to the pattern observed at 8:00 AM, there was not nearly as much co-localization as had been observed at the earlier time point (Figure 9F). The two populations of cells appeared to be more distinct, and many of the Mel1c-immunoreactive cells were overlying the red Mel1a cells, and sometimes appeared to be attached to neighboring Mel1a-immunoreactive cells, thus showing some co-localization (yellow). There appeared to be more Mel1a and Mel1c cytoplasmic labeling at 12:00 N than at 8:00 AM, but the cytoplasmic immunolabeling of the two receptors was not co-localized.

At 4:00 PM (10 h after lights on), most of the Mel1a and Mel1c immunolabeling was co-localized on the lateral membranes, with some cytoplasmic labeling not co-localized (Figure 9G-I). Also, the fluorescent intensity of both Mel1a and Mel1c immunolabeling was much higher than at the previous time points, although this is not demonstrated in the figure panels, because the images would have been over-saturated if presented at the equivalent intensity settings as in the previous time points.

**Figure 10:**
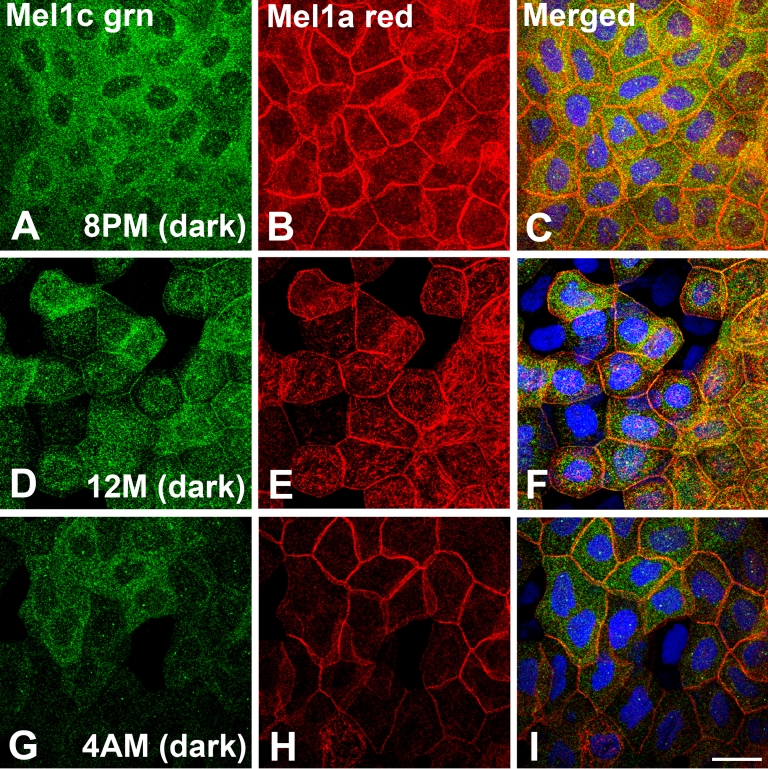
Mel1a and Mel1c immunocytochemistry of whole-mounted *Xenopus laevis* surface corneal epithelium obtained at 4-h intervals during a 24-h light–dark cycle. Frogs were housed under a 12 h:12 h light–dark cycle (6:00 AM: lights on; 6:00 PM: lights off). All tissues in this figure were obtained in the dark. Mel1c labeling is represented in green (**A**, **D**, and **G**) and Mel1a labeling is represented in red (**B**, **E**, and **H**). The yellow labeling in the merged images (**C**, **F**, and **I**) indicates regions of co-localization of the red and green signal. **A-C**: Corneas obtained at 8:00 PM (2 h after lights off). Mel1c immunolabeling is almost exclusively located in the cytoplasm, but there is intense Mel1a immunoreactivity present in the lateral membranes, and also in the cytoplasm. The cytoplasmic immunolabeling of Mel1a and Mel1c is not co-localized. **D-F**: Corneas obtained at 12:00 M (mid-dark). Most of the Mel1c immunoreactivity is in the cytoplasm, although some lateral membrane labeling is also detected. Mel1a immunoreactivity is predominant in the lateral membranes, but there are many irregular-appearing cytoplasmic compartments that express Mel1a immunoreactivity, and they do not co-localize with the Mel1c cytoplasmic labeling. Essentially, all Mel1c lateral membrane labeling is co-localized with Mel1a membrane labeling. **G**-**I**: Corneas obtained at 4:00 AM (2 h before lights on). Most of the Mel1c immunoreactivity is located in the cytoplasm, with very little membrane labeling detected. Most of the Mel1a immunoreactivity is located on the lateral membranes, with some immunoreactivity also appearing in irregular cytoplasmic compartments. The Mel1a and Mel1c cytoplasmic labeling is not co-localized. Nuclei are stained with DAPI. The confocal images in all panels are comprised of three optical slices of 400 nm each in the z-series. The magnification bar (**I**) represents 20 µm.

At 8:00 PM (2 h after lights off), Mel1c immunolabeling was almost exclusively located in the cytoplasm (Figure 10A), whereas there was intense Mel1a immunoreactivity present in the lateral membranes and also in the cytoplasm (Figure 10C). The cytoplasmic immunolabeling of Mel1a and Mel1c was not co-localized (Figure 10C).

At 12:00 M (midnight; middle of the dark period), most of the Mel1c immunoreactivity remained in the cytoplasm, although some lateral membrane labeling was also detected (Figure 10D). Mel1a immunoreactivity was still predominant in the lateral membranes, but there were many irregular-appearing cytoplasmic compartments that expressed Mel1a immunoreactivity, and these structures did not co-localize with the Mel1c cytoplasmic labeling (Figure 10F). Essentially, all the Mel1c lateral membrane labeling co-localized with the Mel1a membrane labeling (Figure 10F).

At 4:00 AM (10 h after lights off), most of the Mel1c immunoreactivity was located in the cytoplasm, with very little membrane labeling detected (Figure 10G). In contrast, most of the Mel1a immunoreactivity was located on the lateral membranes, with some immunoreactivity also appearing in the irregular cytoplasmic compartments (Figure 10H). The Mel1a and Mel1c cytoplasmic labeling was not co-localized (Figure 10I).

**Figure 11:**
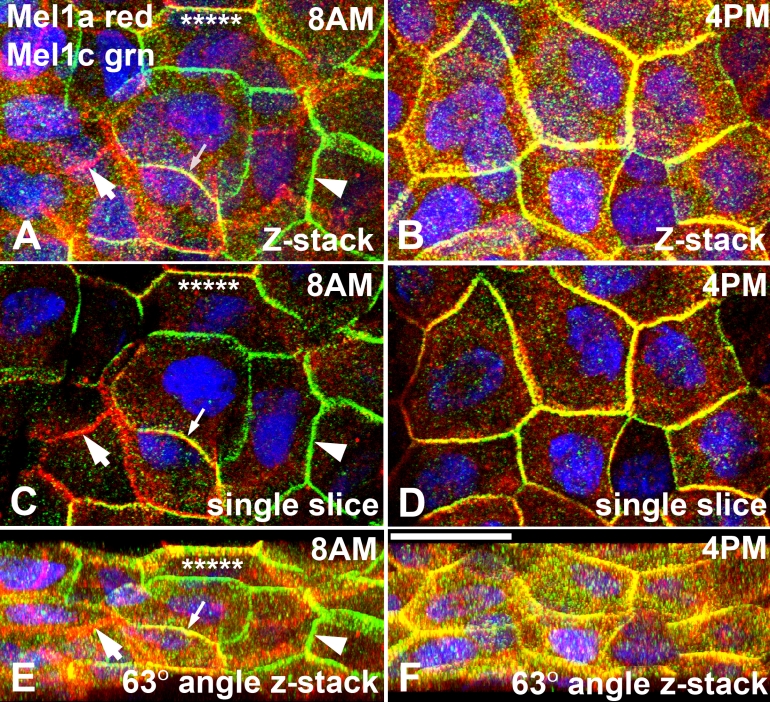
Confocal analysis of Mel1a and Mel1c immunocytochemistry of whole-mounted *Xenopus laevis* surface corneal epithelium at two separate time points. Three-dimensional reconstructions of confocal z-stacks of optical slices of the 8:00 AM (**A**, **C**, and **E**) and 4:00 PM (**B**, **D**, and **F**) specimens were rotated at 63° on the x-axis to enable optimal viewing of the pattern of immunolabeling. At 8:00 AM (**A**, **C**, and **E**), areas of lateral membranes express only the red Mel1a receptor label (large arrow) or the green Mel1c receptor label (large arrowhead). In some areas of merged Mel1a­-Mel1c co-localization, only the yellow color is observed (small arrow), indicating receptor co-localization. There are also areas of membrane that express the yellow co-localization but have small areas of red Mel1a or green Mel1c label interdigitated with the yellow label (asterisks). At 4:00 PM (**B**, **D**, and **F**), the lateral membranes show a more uniform pattern of immunolabeling than observed at the 8:00 AM time point. The yellow co-localization label is predominant in the most apical portion of the membranes, but in the more basal area of the lateral membranes distinct punctate red Mel1a and green Mel1c labeling is also observed. Nuclei are stained with DAPI. The confocal images in panels **A** and **E** are comprised of 16 optical slices of 400 nm each in the z-series. The confocal images in panels **B** and **F** are comprised of 16 optical slices of 400 nm each in the z-series. The images in panels **C** and **D** are comprised of a single optical slice of 400 nm. The magnification bar (**F**) represents 20 µm.

A more detailed analysis was performed on the two time points that demonstrated the greatest differences in receptor localization. The 8:00 AM time point showed that some areas of lateral membranes of some cells expressed only Mel1a or only Mel1c labeling, whereas other parts of the lateral membranes expressed both Mel1a and Mel1c (Figure 9C). In contrast, at the 4:00 PM time point, almost all Mel1a and Mel1c lateral membrane immunolabeling was co-localized (Figure 9I). Three-dimensional reconstructions of confocal z-stacks of optical slices of the 8:00 AM and 4:00 PM specimens were rotated at 63° on the x-axis to enable optimal viewing of the pattern of immunolabeling. At 8:00 AM, areas of the lateral membranes expressed only the red Mel1a receptor label or the green Mel1c receptor label (Figure 11). In areas of membrane that expressed both red Mel1a and green Mel1c labeling, there appeared to be two different patterns. In some areas of merged Mel1a-Mel1c co-localization only the yellow color was observed, indicating such a close proximity of the red Mel1a and green Mel1c labels that it exceeded the level of resolution provided by the methods used in this analysis (Figure 11E). In contrast, there were also areas of membrane that expressed the yellow co-localization, but appeared to have small areas of red Mel1a or green Mel1c label interdigitated with the yellow label (Figure 11E). In contrast to the 8:00 AM time point, the lateral membranes of the 4:00 PM time point showed a much more uniform pattern of immunolabeling. In the most apical portion of the membranes, the yellow co-localization label was predominant. However, in the more basal area of the lateral membranes, distinct punctate red Mel1a and green Mel1c labeling was observed (Figure 11F). This pattern of distinct punctate red Mel1a and green Mel1c labeling on the more basal portion of the lateral membrane was also observed at the 8:00 AM time point (Figure 10E). This suggests that in the more apical areas of the lateral membranes, the Mel1a and Mel1c receptors are in close juxtaposition to each other, whereas in the more basal areas, the Mel1a and Mel1c receptors are not in very close proximity to each other.

## Discussion

The corneal epithelium (CE) is a stratified squamous non-keratinized epithelium. The surface layer of the CE is the only layer of cells that forms tight junctions, which provides the barrier function of the cornea [25-28]. The basal layer of CE cells appears to undergo a circadian rhythm in their rate of proliferation [15-19,29], and as the cells divide, they give rise to daughter cells that are displaced apically. These epithelial cells (termed “wing cells”) continue to be displaced apically as additional cells are generated from the basal epithelium. The addition of new cells from the basal layer is balanced by a loss of epithelial cells at the surface. The surface epithelial cells are desquamated (“shed”) on a daily basis as part of a renewal process [10,11].

We assume that appropriate temporal coordination of the assembly and disassembly of junctional complexes on the surface epithelial cells is necessary for optimal CE function. The expression of melatonin receptors on the surface cells of the CE suggests that melatonin may provide a signal to coordinate some circadian activities of these cells. Melatonin is the major chemical output of the circadian clock, and performs a variety of functions in many tissues [1]. Melatonin is produced by the pineal gland, retinal photoreceptors, and ciliary epithelium on a circadian rhythm, with highest levels produced at night [6,30,31]. The source of melatonin that reaches the CE may therefore be the pineal gland, retina, ciliary epithelium, or a combination of these organs. The purpose of this study was to describe the relative distribution of the melatonin receptor subtypes in *Xenopus laevis* surface CE cells, and to examine the potential changes in sub-cellular receptor distribution with time of day. We report here that the Mel1a, Mel1b, and Mel1c receptors are located on the lateral membranes in the surface layer of cells in *Xenopus* CE, and that their relative distribution on the lateral membranes and cytoplasmic compartments oscillates during a 24-h period. Furthermore, the Mel1a receptor is closely juxtaposed to the zonula occludens protein ZO-1, which may reflect a functional relationship between the receptor and the CE tight junctions.

Previous studies have demonstrated that the Mel1a, Mel1b, and Mel1c receptors are present in the CE of *Xenopus laevis* and the chick [7,8,21], and the MT1 receptor is expressed in human CE [9]. The MT1, MT2, and orphan G protein-coupled receptor 50 (GPCR50) receptors are the mammalian orthologs of Mel1a, Mel1b, and Mel1c, respectively [32,33]. We chose to use *Xenopus* as the animal model for these studies since it has been a popular animal model in the study of the role of melatonin and circadian rhythms in ocular tissues [2,34,35], and the antibodies that we have produced against the three melatonin receptor subtypes are specific for the receptor sequences in *Xenopus*.

In an attempt to further our understanding of the potential for regional variations in *Xenopus* surface CE morphology, we analyzed hematoxylin and eosin (H&E)-stained corneal paraffin sections generated in a previous publication on *Xenopus laevis* histology [36]. We observed that, contrary to a commonly held assumption that *Xenopus laevis* do not have eyelids, they do indeed have a lower eyelid [36]. Moreover, the inferior region of the cornea that is potentially covered by this lower eyelid has a thinner CE than does the superior cornea that is not covered by the eyelid (data not shown). The CE of the inferior cornea is about 30% thinner than the CE of the superior cornea due to about two fewer cell layers near the corneal surface. This suggests that the gentle abrasion caused by eyelid blinking facilitates CE cell desquamation, as is commonly assumed. This feature of the *Xenopus* cornea may provide a uniquely valuable model insofar as the influence of eyelid blinking on the rate of CE desquamation can be accounted for in future studies on the potential role of melatonin signaling in CE turnover.

### Relative distribution of Mel1a and Mel1c in the corneal surface epithelium

The relative locations of the Mel1a and Mel1c receptor subtype immunoreactivity in the corneal surface epithelium were examined in preparations of whole cornea obtained during the mid-light period (12:00 N; Figure 3). Most of the Mel1a (red) and Mel1c (green) immunoreactivity is localized to the lateral plasma membranes, with some punctate immunolabeling also occurring in the cytoplasm. The Mel1a and Mel1c punctate labeling that occurs in the cytoplasm is not co-localized, suggesting that the two receptors are located in separate cytoplasmic compartments. When stacks of the confocal optical slices are viewed at an angle of 72°, it becomes obvious that the Mel1a receptors are located directly apically to the Mel1c receptors (Figure 3B). This is an unanticipated and interesting observation, and suggests that there may be a mechanism by which the two receptor subtypes are tethered to maintain a precise proximity to each other. However, the relative lack of merged yellow fluorescence does not support the concept of receptor heterodimerization in this instance. The lack of support for Mel1a-Mel1c receptor heterodimerization is confirmed by the observation that serial optical slices show a transition from only red Mel1a labeling at the apical position to only green Mel1c labeling at the more basal position (Figure 4). It should be emphasized that this analysis applies only to the mid-light time point, and as discussed below, there is support for the concept of potential Mel1a-Mel1c receptor heterodimerization at other times of day (Figure 11).

### Relative distribution of Mel1a and Mel1b in the corneal surface epithelium

Double-labeling immunocytochemistry of Mel1a and Mel1b receptors shows a distribution pattern that is significantly different from the Mel1a-Mel1c labeling pattern. In corneal specimens obtained at the mid-light period (12:00 N), the lateral membranes of the corneal surface epithelium display a high level of immunoreactivity for both receptor subtypes, as indicated by the yellow fluorescence of the merged red and green images (Figure 5). When analyzed in a manner identical to that described above for the Mel1a-Mel1c double-labeling, a broad band of yellow immunolabeling is observed on the lateral membranes (Figure 5B). Punctate cytoplasmic immunolabeling of the Mel1a and Mel1b receptors is not co-localized, indicating that the receptors are located in separate cytoplasmic compartments. Some punctate red Mel1a immunolabeling is also observed on the lateral membranes located basal to the major yellow fluorescence, indicating that not all the Mel1a on the lateral membrane is in close proximity to the Mel1b receptor (Figure 5A). There is also some red Mel1a labeling interdigitating with the broad yellow band of fluorescence, suggesting that although much of the membrane-associated Mel1a receptor is in very close proximity to the membranous Mel1b receptors, a significant amount of membranous Mel1a receptor is not as closely associated (Figure 5B). In contrast to the gradual transition from red Mel1a to green Mel1c membrane labeling in serial optical slices (Figure 3), all serial optical slices of the Mel1a-Mel1b labeling, representing 2 µm in thickness, contain mostly yellow fluorescence, with some red and a very small amount of green fluorescence interposed (Figure 6). Taken together, the receptor double-labeling experiments suggest that the Mel1a and Mel1b receptors are located at approximately the same apical/basal position on the lateral membranes, and the Mel1c receptor is located directly basal to that location.

### Relative distribution of Mel1a and ZO-1 in the corneal surface epithelium

Double-label Mel1a-ZO-1 immunocytochemistry was performed in an attempt to determine the location of the melatonin receptor complex relative to tight junctions. ZO-1 is a zonula occludens protein associated with cytoplasmic plaques of tight junctions, and it interacts with a wide variety of cellular proteins and plays a central role in orchestrating tight junction complexes [37-39]. The utility of ZO-1 as an excellent marker for tight junctions is not limited to our interest in the spatial relationship of the melatonin receptors to tight junctions, but is also valuable for evaluation of the potential role of melatonin receptor signaling in rhythmic tight junction assembly. ZO-1 is primarily localized to a relatively narrow strip of labeling on the lateral membranes (Figure 7), although punctate ZO-1 immunolabeling is often observed in the cytoplasm, as expected (Figure 8). The apical to basal width of the band of ZO-1 immunoreactivity is significantly smaller than observed for the band of Mel1a immunoreactivity (Figure 7), indicating that Mel1a (and by inference, Mel1b and Mel1c) distribution is more widespread on the lateral membrane than are the tight junctions.

At the late afternoon time point (4:00 PM) that was selected for this analysis, Mel1a immunoreactivity is dispersed along the entire lateral membrane that is located basal to the tight junctions (Figure 7). It is interesting to note that Mel1a receptor immunoreactivity is generally lacking in surface CE cells that have small apical profiles (Figure 7 and Figure 8). These small profiles presumably indicate the limits of the lateral membranes of CE cells that have just recently emerged at the surface layer, and are therefore relatively immature compared to the larger surface cells. From this observation, we surmise that tight junction formation precedes Mel1a expression in the lateral membranes of the surface epithelium. However, we also occasionally observed some larger mature cells expressing Mel1a receptor on the lateral membranes, but not expressing ZO-1 immunoreactivity (Figure 7 and Figure 8). ZO-1 expression on lateral membranes may become diminished in mature cells, perhaps as part of the desquamation process. It is known that tight junction proteins pre-accumulate in the sub-superficial cells prior to desquamation of the surface cells [12,13,40], so any potential correlation between Mel1a expression and ZO-1 expression on the surface cells would not be indicative of a potential role for melatonin signaling in tight junction formation.

Analysis of the relative distribution of Mel1a to ZO-1 compared to the localization of Mel1a relative to Mel1c reveals some interesting features of melatonin receptor membrane localization. Surprisingly, Mel1a appears to be more closely associated with ZO-1 than with Mel1c. As described earlier, the Mel1a (red) labeling appears as a discrete band directly apical to a band of Mel1c (green) labeling, with a small amount of overlap between the red and green fluorescence that results in a merged yellow signal (Figure 3 and Figure 4). In contrast, when ZO-1 and Mel1a are both expressed on CE lateral membranes, a discrete band of green ZO-1 is not observed, and a broad red Mel1a band is observed just basal to the thinner ZO-1-Mel1a merged yellow signal (Figure 7 and Figure 8). Therefore, ZO-1 is in such close proximity to Mel1a that a discrete apical ZO-1 (green) signal is not detected, yet Mel1c is far enough away from Mel1a to enable us to detect an apical red Mel1a signal that is distinct from the green and yellow signals. The exception to this pattern is a discrete green ZO-1 band observed in immature surface CE cells in which Mel1a is not yet expressed (Figure 7 and Figure 8). It is tempting to speculate that the relatively close association of Mel1a with ZO-1 is a reflection of a functional association between these proteins. There is an emergent realization that G protein-coupled receptor (GPCR) signaling depends to some extent upon association with organized networks of scaffolding proteins that optimize the specificity and timing of the cellular responses. Many GPCRs interact with the postsynaptic density protein 96 (PSD-96)/*Drosophila* Disc large/ZO-1 homology (PDZ) domain-containing proteins which modulate receptor signaling by assembling the different proteins involved in the transduction of the signal to the target proteins [41-44]. For example, the multi-PDZ domain protein (MUPP1) interacts with the COOH-terminal tail of mammalian Mel1a (MT1) to promote Gi coupling and signaling of the MT1 receptor [42]. Interestingly, MUPP1 is concentrated at tight junctions through interactions with junctional proteins such as claudin-1 and junctional adhesion molecule (JAM) [45], and the PDZ domains of ZO-1 bind directly to the COOH-terminus of claudin [46]. These observations suggest a molecular architectural model in which GPCRs are tethered to macromolecular complexes which include tight junction proteins by PDZ domain scaffolding proteins. The recent observation that the GPCR somatostatin receptor 3 interacts with MUPP1 to control epithelial tight junction permeability suggests that perhaps other MUPP1-associated GPCRs such as the melatonin receptors could potentially contribute to regulation of tight junctions [47].

### Diurnal rhythm of Mel1a and Mel1c receptor localization

Diurnal oscillations in melatonin receptor RNA expression, protein expression, and binding sites have been reported in a variety of tissues and species [8,48-53]. It has been suggested that the rhythms in melatonin receptor expression may be superimposed on the circadian rhythm in melatonin synthesis as an additional level of regulation of melatonin signaling [54]. Certainly, the availability of functional binding sites would be expected to have a profound impact on the cellular responses to ligand exposure. In addition to potential regulation of melatonin receptor availability by alterations in receptor RNA and/or protein synthesis, receptor availability can also potentially be regulated by receptor sequestration/degradation in intracellular compartments following ligand binding and receptor activation. Furthermore, homodimerization, heterodimerization, and association with macromolecular complexes can also affect GPCR receptor activity [42,47,55-58].

To assess the potential for daily changes in melatonin receptor expression or localization in *Xenopus* CE, we performed a double-label immunocytochemical analysis of Mel1a and Mel1c receptors in whole corneas obtained at 4-h intervals over a 24-h period. The 8:00 AM time point (2 h after lights on) shows a distinctive pattern in which only Mel1a, only Mel1c, and merged Mel1a-Mel1c labeling is present in different regions of the surface CE lateral membranes (Figure 9C). It appears that the region of membrane contact between neighboring cells displays a uniform pattern of Mel1a and/or Mel1c receptor expression, but when the membrane contact changes to contact with a different neighboring cell, the pattern of receptor labeling can change. In some membranes that display the yellow Mel1a-Mel1c receptor labeling, it appears that co-localization may be due to expression of only Mel1a on the cell membrane of one cell, and only Mel1c on the cell membrane of the neighboring cell (Figure 11A,C). However, in other areas that display the Mel1a-Mel1c merged yellow labeling, it appears to be the result of both Mel1a and Mel1c expression on both membranes. These observations support the possibility that heterodimerization of Mel1a and Mel1c exists in some, but not all, CE lateral membranes at the 8:00 AM time point. Further studies are needed to assess the relative degrees of homodimerization and heterodimerization of melatonin receptors at different times of day on the corneal surface epithelium.

In contrast to the diverse Mel1a-Mel1c labeling pattern observed at 8:00 AM, the 4:00 PM pattern of Mel1a and Mel1c labeling is consistently co-localized (Figure 9 G-I). Furthermore, discrete labeling of only Mel1a or only Mel1c could not be readily discerned (Figure 11F), indicating that the two receptors are in very close proximity to each other in the majority of surface cells. Whether the different patterns of relative Mel1a and Mel1c labeling observed at the 8:00 AM and 4:00 PM time points represent daily changes in receptor dimerization cannot be determined from this study, but it remains a possibility that is worthy of further study. These results do suggest that there are daily changes in melatonin receptor subtype localization on the surface CE lateral membranes, with the highest degree of Mel1a-Mel1c co-localization occurring late in the light period. The punctate receptor immunolabeling that occurs in the cytoplasm during the latter part of the light period may be the result of transport vesicles carrying newly-synthesized receptors to the plasma membrane. Previous reports demonstrating a diurnal rhythm of melatonin receptor synthesis during the day support this possibility [8,48-53].

Recent studies have demonstrated that most GPCRs interact with each other to form dimers and/or oligomers, and that this is essential for their activation [55,56,59]. GPCRs have the ability to heterodimerize, and these heterodimers exhibit distinct functional properties [56-58,60]. Dimerization of GPCRs appears to be a universal phenomenon that provides an additional mechanism for modulation of receptor function as well as cross-talk between GPCRs [59]. Mammalian MT1 and MT2 melatonin receptors (equivalent to Mel1a and Mel1b, respectively) can exist as homodimers and as heterodimers, and the relative expression and affinities of each receptor subtype may determine the proportion of homodimers and heterodimers [61]. GPR50 is an orphan GPCR that has been recently discovered to be the mammalian ortholog of Mel1c [33], and to heterodimerize with MT1 and MT2 melatonin receptors [58]. Heterodimerization with GPR50 decreases the function of the MT1 receptor because of an interaction of the C-terminal tail of GPR50 with regulatory proteins of MT1 receptors, whereas heterodimerization between GPR50 and MT2 does not modify MT2 function [58]. Our observation of a transient close association of the Mel1a and Mel1c receptors in frog CE provides in vivo support of previous reports of [48] recombinant MT1-GPR50 heterodimerization in mammalian cell lines. Further studies are planned to investigate the potential role of melatonin receptor heterodimerization on melatonin signaling in *Xenopus* CE.

At 8:00 PM (2 h after lights off), Mel1a labeling is prominent in the lateral membranes, but most of the Mel1c labeling is present in small punctate cytoplasmic compartments (perhaps endosomes) and there does not appear to be any co-localization of the two receptor subtypes (Figure 10A-C). One explanation for this translocation of Mel1c immunolabeling from the lateral membrane to the cytoplasm is endocytosis of the activated receptors. Interestingly, some Mel1a receptor is translocated to large, irregular cytoplasmic compartments four hours later at 12:00 M (Figure 10E). The Mel1a-containing irregular cytoplasmic compartments may represent late endosomes. These observations suggest that the Mel1c receptor is activated and internalized earlier in the dark period than the Mel1a receptor. Receptor internalization and down-regulation is common in GPCRs, and some melatonin receptors appear to be regulated by this mechanism [62-65]. Melatonin differentially regulates MT1 and MT2 (Mel1a and Mel1b, respectively) receptors in transfected mammalian cell lines. MT2, but not MT1, is rapidly internalized through an arrestin-dependent mechanism after exposure to melatonin [62,63]. Long-term exposure to high concentrations of melatonin desensitizes recombinant MT1 receptors in transfected cells without inducing internalization [64]. However, it has been recently shown that chronic exposure (5 h) of MT1-transfected cells results in receptor internalization and β-arrestin binding [65]. The binding of β-arrestin to the presumably phosphorylated MT1 in endosomes promotes scaffolding of mitogen-activated protein kinase/extracellular signal-regulated kinase 1/2 (MEK/ERK 1/2) which leads to signaling via the microtubule-associated protein (MAP) kinase pathway. This suggests that melatonin-induced endocytosis of the receptor may promote intracellular signaling when the receptors are sequestered in an endosomal compartment. There is strong evidence that endocytosis of some GPCRs is required for activation of specific signal transduction pathways such as the MAP kinase cascade, suggesting that active signaling events occur during the receptor transit in endosomal compartments [66-70]. Together, these findings support the concept that melatonin receptor subtypes are differentially regulated, which can result in different temporally-regulated cellular responses to nocturnal melatonin exposure.

Potential changes in receptor heterodimerization during the diurnal cycle may also play an important role in regulating the availability of the various receptor subtypes to ligand binding. Heterodimerization can promote or inhibit the co-internalization of both receptors after stimulation of just one of the protomers [56]. For example, recombinant MT1 homodimers are internalized in response to melatonin stimulation, whereas GPR50 homodimers and MT1-GPR50 heterodimers are not internalized in response to melatonin [58]. This suggests that stimulation of the GPR50 protomer prevents the internalization of the MT1-GPR50 heterodimer. Based on the observations on the diurnal changes in melatonin receptor location, we suggest this potential scenario: (1) Mel1a and Mel1c are synthesized and transported via transport vesicles to the lateral membranes during the light period, (2) during the light period, some of the Mel1a and Mel1c monomers form heterodimers on the lateral membrane, although some remain as monomers or homodimers, (3) in the dark, when melatonin levels are beginning to rise, Mel1c receptors are stimulated and are internalized into endosomes (which would require dissociation from Mel1a if present as Mel1a-Mel1c heterodimers), (4) later in the dark period, as melatonin levels continue to rise, the Mel1a receptors are stimulated and some are internalized, eventually being delivered to late endosomes, (5) as melatonin levels decline in the late dark period and early light period, melatonin receptors are degraded or recycled back to the lateral membrane, and new receptors begin to be synthesized. This scenario is highly speculative and requires further study to confirm or refute this possibility.

There is support for the concept of internalization of a single ligand-activated protomer that has dissociated from a receptor heterodimer, as previously reported for somatostatin receptor (sst) heterodimers. Somatostatin induces endocytosis of sst_2A_ and sst_3_ homodimers, but sst_2A_-sst_3_ heterodimers dissociate at the plasma membrane, and only sst_2A_, but not sst_3_, undergoes ligand-induced endocytosis after exposure to somatostatin [71]. This supports our speculation that melatonin binding to the Mel1a-Mel1c heterodimer induces internalization of the Mel1c, but not the Mel1a, protomer early in the dark period (Figure 10A,B). Furthermore, the separation of Mel1c from Mel1a may potentially increase the sensitivity of the Mel1a that remains on the plasma membrane [58]. Models for ligand-promoted regulation of GPCR heterodimerization are supported by strong experimental evidence [56]. Several studies suggest that ligand binding can regulate heterodimer formation by either promoting or inhibiting dimerization [41,72-75]. In this model, GPCR monomers and/or heterodimers on the plasma membrane may respond to ligand binding by monomer dimerization and/or dimer dissociation. Dissociated monomers could then potentially dimerize with other monomers on the membrane.

In conclusion, we have observed that Mel1a, Mel1b, and Mel1c melatonin receptor immunoreactivity is present on the lateral membranes and in cytoplasmic compartments of the *Xenopus laevis* corneal surface epithelium. Mel1a is located basal to the zonula occludens protein, ZO-1, and the very close proximity of melatonin receptors to the tight junctions supports a potential role for melatonin in CE barrier function. Mel1a and Mel1b are closely associated with each other on the lateral membrane, but Mel1c is generally located in a basal position to Mel1a, although in close proximity during the mid-light period. The relative positions of the Mel1a and Mel1c receptors appear to undergo dynamic changes during the light–dark cycle, insofar as they may co-localize late in the day, and then perhaps dissociate following melatonin binding at night. Cytoplasmic melatonin receptor immunoreactivity varies throughout the day, and may represent melatonin receptor trafficking in transport vesicles and endosomes. The expression of melatonin receptors on the corneal surface epithelium supports the concept of a role for circadian signaling on corneal epithelial function.
